# HIV, Hepatitis C, and Hepatitis B Infections and Associated Risk Behavior in Injection Drug Users, Kabul, Afghanistan

**DOI:** 10.3201/eid1309.070036

**Published:** 2007-09

**Authors:** Catherine S. Todd, Abdullah M.S. Abed, Steffanie A. Strathdee, Paul T. Scott, Boulos A. Botros, Naqibullah Safi, Kenneth C. Earhart

**Affiliations:** * University of California, San Diego, La Jolla, California, USA; † Ministry of Public Health, Kabul, Afghanistan; ‡ Walter Reed Army Institute of Research, Rockville, Maryland, USA; § United States Naval Medical Research Unit 3, Cairo, Egypt

**Keywords:** HIV, Afghanistan, injection drug users, hepatitis C, hepatitis B, needle sharing, research

## Abstract

Limited prevalence data for HIV, hepatitis B surface antigen (HBsAg), and hepatitis C virus (HCV) exist for Afghanistan. We studied a cross-sectional sample of adult injection drug users (IDUs) in Kabul, Afghanistan, from June 2005 through June 2006. Study participants completed interviewer-administered questionnaires and underwent testing for HIV, antibody to HCV, and HBsAg. Overall prevalences of HIV, HCV, and HBsAg were 3.0% (95% confidence interval [CI] 1.7%–5.1%), 36.6% (95% CI 32.2%–41.0%), and 6.5% (95% CI 4.2%–8.7%), respectively (N = 464). Among male IDUs (n = 463), risky behavior, including sharing syringes (50.4%), paying women for sex (76.2%), and having sex with men or boys (28.3%), were common. Needle sharing, injecting for >3 years, and receiving injections from nonmedical providers were independently associated with increased risk for HCV infection. The high prevalence of risky behavior indicate that Kabul is at risk for an HIV epidemic. Scale-up of harm-reducing interventions is urgently needed.

## Abstract 2

Behavior of injection drug users increases the risk for an HIV epidemic.

Injection drug use has become increasingly common in Central and South Asia, fostered by readily available opium and heroin (*1*,*2*). Many countries in this region are experiencing HIV epidemics driven by injection drug use that is extending to other populations (*3*,*4*). Four countries bordering Afghanistan (Pakistan, Tajikistan, Uzbekistan, and Iran), which provided refuge to many Afghans during the extended period of civil war, are experiencing HIV epidemics among injection drug users (IDUs) (*1*,*3*,*5*,*6*). The population of Kabul, the capital of Afghanistan, has increased to ≈3 million since 2001 because of returning refugees (*7*,*8*). Refugees may have acquired high-risk bebavior, such as injection drug use, that may place communities at risk upon their return.

Afghanistan provided 88% of the world’s opium supply in 2005 (*9*). Although noninjection use of opium (smoking, vaporization, or oral ingestion) is traditional in Afghanistan, injecting likely represents a new behavior (*10*). This behavior may be learned in countries of refuge during times of political unrest, as indicated by the participants in a United Nations Office on Drugs and Crime study in 2003, in which 50% (n = 34) of participants had started using heroin in either Pakistan or Iran (*11*). A prior study in the border city of Quetta, Pakistan, reported that Afghan IDUs were more likely than their Pakistani counterparts to engage in risky behavior (*12*). These observations raised concern that injection drug use and accompanying high-risk behavior are increasing in Afghanistan and that a concentrated HIV epidemic may soon ensue (*13*).

There were an estimated 470 IDUs in Kabul in 2003, although the United Nations Office on Drugs and Crime Afghanistan survey in 2005 estimated that there were 50,000 heroin users in Afghanistan, of whom 14% reported injecting drugs (*10*,*11*). The same study also estimated that most IDUs reside in Kabul and, of all heroin-using IDUs interviewed, 70% stated they had shared needles (*10*). Of IDUs interviewed, all were men, but anecdotal evidence from harm-reduction programs indicated that a few IDUs in Kabul were female (*10*). Although drug use is illegal in Afghanistan and warrants either rehabilitation for a first offense or imprisonment for recurrent offenses (*14*) the motivating factor stated for initiating injection was constant pain that was not relieved by smoking (*10*). However, little is currently known about other aspects of injection drug use in Kabul, such as syringe sources or harm-reducing programs.

Little data are available on HIV, hepatitis B surface antigen (HBsAg), or hepatitis C virus (HCV) prevalence and associated risk behavior in Afghanistan. As of October, 2005, only 41 cases of HIV had been reported, although this is believed to underestimate the potential problem (*15*). We assessed prevalence of HIV, HCV, HBsAg, and associated risk behavior among IDUs in Kabul.

## Methods

### Study Design and Participants

We conducted a cross-sectional study of IDUs in Kabul, Afghanistan, from June 2005 through June 2006, through the Voluntary Counseling and Testing (VCT) Center at the Central Polyclinic, an Afghan Ministry of Public Health facility. At the time of this study, there were 3 harm-reduction programs in Kabul, of which 1 had on-site syringe exchange.

Eligible participants were those >18 years of age who reported having injected drugs within the past 6 months (confirmed through injection marks) and were able to provide informed consent. Before data collection, this study was reviewed and approved by the investigational review boards of the University of California, San Diego; the US Naval Medical Research Unit No. 3; the Walter Reed Army Institute of Research; and the Ministry of Public Health of Afghanistan.

### Procedures

Potential participants were approached by an experienced outreach worker known to them. If participants were interested in entering the study, they accompanied the outreach worker to the VCT Center. At the VCT, a study representative explained the study in a confidential setting and obtained informed consent. The participant was assigned a unique study number, the sole identifier, which was required for receiving test results if required. Participants were interviewed by a trained study representative matched to the participant’s sex. The questionnaire included sociodemographics, travel and medical histories, past and current drug use and sexual behavior, and knowledge of bloodborne and sexually transmitted infections. No data were recorded from those declining participation or ineligible to enter the study.

Pretest and posttest counseling were given, and rapid antibody testing was performed by using the Abbott Determine HIV 1/2 test, the Abbott Determine HBsAg test (both from Abbott Diagnostics Japan, Tokyo, Japan), and the Standard Diagnostics HCV test (Standard Diagnostics Laboratories, Yongin-si Gyeonggi-do, Republic of Korea) for HCV. Participants with a positive HIV test result underwent sequential testing with the OraSure OraQuick HIV 1/2 test (OraSure Technologies, Bethlehem, PA, USA). Repeatedly positive rapid HIV test results were confirmed by Western blot (HIV BLOT 2.2; GeneLabs Diagnostics, Singapore). Hepatitis B was confirmed with a second, serum-based rapid test (Standard Diagnostics HBV; Standard Diagnostics Laboratories) because nucleic acid testing was not available in Kabul. The Abbott and Standard Diagnostics HBsAg rapid tests had sensitivities of 99.0% and 99.0% and specificities of 99.0% and 100.0%, respectively, and a positive predictive value of 99.9%, assuming a baseline HBsAg prevalence of 5.0% (*16*,*17*). The presence of antibody to HCV was confirmed with a recombinant immunoblot assay (RIBA) test (RIBA 3.0 SIA; Chiron Corporation, Emeryville, CA, USA).

All confirmatory testing was performed at the VCT Center in Kabul by trained laboratory personnel. All participants received a small nonmonetary gift and risk-reduction counseling, with referrals for detoxification and needle and syringe programs upon request.

### Statistical Analysis

Prevalence of infection was calculated with confidence intervals (CIs) based on Poisson distribution for HIV and binomial distribution for HBsAg and HCV. The only female participant was excluded from remaining analyses. Correlates of HIV, HBsAg, and HCV infection were assessed with univariate and multivariate logistic regression analyses. Variables were entered into a multivariate model if they were significantly associated with HIV, HBsAg, or HCV infection at the 5% level in univariate analysis or showed epidemiologic relationships. A multivariate model was generated to identify factors independently associated with HIV, HBsAg, and HCV infections by using the likelihood ratio test to determine which variables were retained.

## Results

### Sociodemographic Data and Prevalence of Infection

A total of 464 participants were enrolled; 463 were male. Fourteen participants (3.0%, 95% CI 1.7%–5.1%) were infected with HIV, 30 (6.5%, 95% CI 4.2%–8.7%) were positive for HBsAg, 170 (36.6%, 95% CI 32.2%–41.0%) were infected with HCV, and 7 (1.5%, 95% CI 0.6%–3.1%) were coinfected with HIV and HCV.

Among male participants, most were Afghan, had traveled outside Afghanistan in the previous 10 years, and reported heroin as their most frequently used drug in the past 6 months, either alone (42.4%) or with pheniramine maleate (56.0%) (Appendix Table).

### Risk Behavior

High-risk injection and sexual behavior were common. Sharing needles or syringes (50.4%) and difficulty obtaining new syringes (43.6%) were frequently reported. Patronizing female sex workers and having sexual relations with men or boys were also common. More than half the participants had been incarcerated; of these, nearly one third injected drugs while in prison. A total of 23.1% had received a therapeutic injection in the past 6 months, and 5.2% had sold or donated blood (Appendix Table).

### Correlates of HIV, HBsAg, and HCV Infection

No sociodemographic variables were significantly associated with HIV or HBsAg infection. Sharing needles and injecting drugs while in prison were associated with HBsAg by univariate logistic regression analysis (Appendix Table). Multivariable analysis showed that HBsAg remained associated with injecting drugs in prison (adjusted odds ratio 3.23, 95% CI 1.16–9.00). Univariate analysis showed that those with HIV infections were more likely to report needle or syringe sharing and injecting drugs for >3 years (Appendix Table). No variables were independently associated with HIV infection by multivariate logistic regression analysis (results not shown).

Participants with HCV infection were less likely to be educated or married and had higher incomes (Appendix Table). HCV infection was associated with needle or syringe sharing, injecting drugs for >3 years, having sex with men or boys, and receiving injections from a nonmedical provider (Appendix Table). Adjustment by demographic factors did not appreciably change these relationships. Multivariate logistic regression showed that needle or syringe sharing, injecting drugs for >3 years, and receiving injections from a nonmedical provider were independently associated with HCV infection, and inverse associations persisted for higher education level and for being married (Table).

**Table:** Factors independently associated with HCV infection (n = 170) by multivariable analysis in 463 male injection drug users, Kabul, Afghanistan*

Factor	Value
HCV prevalence	107 (36.8)
Demographic factors	
Married	0.60 (0.40–0.92)
Higher educational level	0.51 (0.29–0.88)
Drug practices	
Ever shared needle or syringe	2.60 (1.71–3.96)
Duration injection drug use >3 y	3.28 (2.17–4.96)
Medical encounters	
Injections by a nonmedical provider	2.71 (1.26–5.82)

*HCV, hepatitis C virus. Values are no. (%) or adjusted odds ratio (95% confidence interval). Analysis was adjusted for marital status, educational level, duration of injecting, sharing needles or syringes, and injections by a nonmedical provider.

## Discussion

This report is among the first to describe HIV, HBsAg, and HCV prevalence and risk behavior in Afghanistan. The low HIV prevalence among IDUs in Kabul is not surprising given the short median duration of injection drug use. Although opium has been used for centuries in Afghanistan, our data are consistent with the suggestion that injection drug use is a relatively new behavior in this setting (*10*). Although HIV prevalence was low, 37% were HCV infected, a finding that potentially foreshadows an HIV epidemic caused by risk factors shared by these infections.

Injecting drugs in prison was related to HBsAg and marginally to HIV by univariate analysis, which are similar to findings in Iran and other settings (*3*,*18*,*19*). In Iran, Zamani et al. reported that that multiple incarcerations increased likelihood of HIV infection (*20*). We did not observe this relationship, but this result may be due to low statistical power. However, 47% of the Iranian prison population is incarcerated because of drug-related offenses (*18*). Afghan law allows an addict, as diagnosed by a medical professional, to enter a detoxification facility, which may reduce the number and exposure of IDUs to prison settings. Because one third of Afghan IDUs who had been incarcerated reported drug injection in prisons, prisons should remain a priority site for needle and syringe programs..

The established risk factors of needle sharing and duration of injection use were strongly related to both HIV and HCV infections. The prevalence of HBsAg was relatively low among the IDUs and close to that reported by International Committee of the Red Cross/Crescent–supported blood banks in 2004. While only those with acute or active hepatitis B would have circulating antigen, the prevalence of hepatitis B in this group still seems comparatively low, given the prevalence of risky behavior. The prevalence of HIV and hepatitis B infection may not have not reached sufficient levels to result in a self-sustaining epidemic; further surveillance is warranted. The prevalence of HBsAg likely underestimates the number of IDUs exposed to hepatitis B. Because hepatitis B infection resolves after the acute phase in 90%–95% of those infected as adults, the actual proportion of IDUs infected may approach or exceed 64.7% of participants (*21*). A more reliable number might be accessed through testing for antibody to HbsAg, which might be used to create a cost-effective vaccination program for this high-risk group.

Although donating blood was not associated with any of the 3 infections, risk for bloodborne infection through iatrogenic routes deserves emphasis because 8.3% of participants who reported donating or selling blood were infected with HIV. A prior report estimated that only 30% of blood donations were screened in Afghanistan (*22*). Furthermore, those infected with HCV were more likely to have had injections from nonmedical providers, which has been linked to a high prevalence of HCV and hepatitis B in neighboring Pakistan (*23*).

Our study has some limitations. Respondent-driven sampling was not possible because of concerns of compromising the identities of IDUs; participants were enrolled by convenience sampling, which may not be representative of IDUs in Kabul. Because risky behavior was assessed by self-reporting, socially desirable responses may have been made. Analysis of factors associated with HIV and HBsAg had low power because of low prevalence of these infections, which potentially masks some associations. Additionally, testing for surface antigen may have underestimated the true prevalence of hepatitis B infection because only those with acute or chronic infections would be detected. Another approach for future studies would be screening for both surface antigen and antibody to HbsAg and offering vaccination to IDUs negative for this antibody.

In summary, although prevalence of HIV and HBsAg is low among IDUs in Kabul, the prevalence of HCV and high-risk behavior are alarmingly high. Political instability, poverty, mobility, and low literacy may also increase vulnerability of IDUs to HIV and other bloodborne or sexually transmitted infections (*13*). During the study, 1 needle and syringe program and 3 drug rehabilitation and counseling programs were operating in Kabul; opioid substitution treatment was not available. Initiation or scale-up of interventions, particularly needle and syringe programs and opioid substitution therapy, are urgently needed to prevent an HIV epidemic among Afghan IDUs. Attempts to prevent or control HIV and other bloodborne infections among IDUs by not providing adequate coverage with harm-reduction programs have been unsuccessful (*24*,*25*). However, settings with outreach programs that linked VCT, needle and syringe programs, and opiate substitution therapies have stabilized HIV prevalence among IDUs at low levels (*25*,*26*). Political support for harm-reduction and HIV awareness campaigns among the Ministries of Counter Narcotics, Public Health, and Religious Affairs is present in Afghanistan; donor attention is urgently needed to expand these efforts to avert an HIV epidemic.

## Supplementary Material

Appendix Table Univariate analysis of characteristics of 463 male injection drug users, Kabul, Afghanistan*
