# Supplementary material for: HIV, Hepatitis C, and Hepatitis B Infections and Associated Risk Behavior in Injection Drug Users, Kabul, Afghanistan
Source: Emerg Infect Dis. 2007 Sep;13(9):1327–31. doi: 10.3201/eid1309.070036 (PMC2857281; doi:10.3201/eid1309.070036)
Supplement: Appendix Table — Univariate analysis of characteristics of 463 male injection drug users, Kabul, Afghanistan* [file 07-0036_appT-s1.pdf]

**Appendix Table.** Univariate analysis of characteristics of 463 male injection drug users, Kabul, Afghanistan*

| Variable, No. (%) | HIV | | HBsAg | | HCV | |
|---|---|---|---|---|---|---|
| | No. (%) | OR, 95% CI | No. (%) | OR, 95% CI | No. (%) | OR, 95% CI |
| Age group, y | | | | | | |
| ≤30, 276 (59.7) | 7 (2.5) | Reference | 16 (5.8) | Reference | 93 (33.7) | Reference |
| >30, 186 (40.3) | 7 (3.8) | 1.50, 0.52–4.36 | 16 (5.8) | 1.32, 0.63–2.79 | 77 (41.4) | 1.40, 0.93–2.09 |
| Mean 30.6, IQR 25–35 | | | | | | |
| Age began injection drug use, y | | | | | | |
| ≤25, 253 (54.6) | 8 (3.2) | Reference | 15 (6.0) | Reference | 95 (37.7) | Reference |
| >25, 210 (45.4) | 6 (2.9) | 0.90, 0.31–2.63 | 15 (7.1) | 1.22, 0.58–2.55 | 75 (35.7) | 0.92, 0.63–1.34 |
| Mean 26.4, IQR 21–30 | | | | | | |
| Nationality | | | | | | |
| Afghan, 458 (98.9) | 14 (3.1) | – | 30 (6.5) | – | 169 (36.9) | 2.34, 0.23–115.8 |
| Other†, 5 (1.1) | 0 | Reference | 0 | Reference | 1 (20) | Reference |
| Education, y | | | | | | |
| ≤8, 370 (80.1) | 13 (3.5) | Reference | 26 (7.0) | Reference | 145 (39.2) | Reference |
| > 8, 92 (19.9) | 1 (1.1) | 0.30, 0.04–2.31 | 4 (4.3) | 0.60, 0.20–1.77 | 25 (26.9) | **0.58, 0.35–0.96** |
| Marital status | | | | | | |
| Married, 239 (51.7) | 7 (2.9) | 0.93, 0.32–2.69 | 16 (6.7) | 1.07, 0.51–2.24 | 76 (31.8) | **0.64, 0.44–0.94** |
| Unmarried‡, 223 (48.3) | 7 (3.1) | Reference | 14 (6.3) | Reference | 94 (42.2) | Reference |
| Employed | | | | | | |
| Yes, 406 (88.1) | 13 (3.2) | 1.78, 0.29–13.9 | 26 (6.4) | 0.87, 0.29–2.59 | 144 (35.4) | 0.61, 0.33–1.13 |
| No, 55 (11.9) | 1 (1.8) | Reference | 4 (7.3) | Reference | 26 (47.3) | Reference |
| Monthly income, Afghanis§ | | | | | | |
| ≤4,500, 312 (67.5) | 10 (3.2) | Reference | 23 (7.4) | Reference | 103 (33.0) | Reference |
| >4,500, 150 (32.5) | 4 (2.7) | 0.82, 0.25–2.66 | 7 (4.6) | 0.61, 0.26–1.46 | 67 (44.4) | **1.62, 1.06–2.46** |
| Lived outside Afghanistan in past 10 y | | | | | | |
| Yes, 400 (86.4) | 13 (3.3) | 2.09, 0.27–16.29 | 23 (5.8) | 0.49, 0.20–1.20 | 146 (36.7) | 1.01, 0.58–1.75 |
| No, 63 (13.6) | 1 (1.6) | Reference | 7 (11.1) | Reference | 23 (36.5) | Reference |
| Duration of injection drug use, y | | | | | | |
| ≥3, 256 (55.4) | 11 (5.3) | **4.76, 1.31–17.29** | 11 (5.3) | 0.70, 0.33–1.51 | 107 (51.7) | **3.31, 2.23–4.91** |
| <3, 206 (44.6) | 3 (1.2) | Reference | 19 (7.4) | Reference | 63 (24.6) | Reference |
| Mean 4.4, IQR 2–6 | | | | | | |
| Use new needle every time | | | | | | |
| Yes, 135 (29.2) | 3 (2.2) | 0.65, 0.18–2.38 | 6 (4.4) | 0.59, 0.23–1.47 | 55 (40.7) | 1.27, 0.84–1.91 |

| | | | | | | |
|---|---|---|---|---|---|---|
| No, 327 (70.8) | 11 (3.5) | Reference | 24 (7.3) | Reference | 115 (35.2) | Reference |
| Needle or syringe sharing | | | | | | |
| Yes, 233 (50.4) | 11 (4.7) | **3.73, 1.03–13.56** | 22 (9.4) | **2.88, 1.25–6.11** | 108 (46.3) | **2.33, 1.58–3.43** |
| No, 229 (49.6) | 3 (1.3) | Reference | 8 (3.5) | Reference | 62 (27.1) | Reference |
| Donated or sold blood | | | | | | |
| Yes, 24 (5.2) | 2 (8.3) | 3.23, 0.68–15.35 | 1 (4.2) | 0.61, 0.08–4.71 | 8 (33.3) | 0.86, 0.31–2.20 |
| No, 439 (94.8) | 12 (2.7) | Reference | 29 (6.6) | Reference | 162 (36.9) | Reference |
| Received therapeutic injections in last 6 mo | | | | | | |
| Yes, 106 (22.9) | 4 (3.8) | 1.36, 0.42–4.42 | 10 (9.4) | 1.75, 0.79–3.86 | 46 (43.4) | 1.43, 0.92–2.23 |
| No, 356 (77.1) | 10 (2.8) | Reference | 20 (5.6) | Reference | 124 (34.8) | Reference |
| Received injections from a nonmedical provider | | | | | | |
| Yes, 35 (7.6) | 1 (2.9) | 0.93, 0.12–7.32 | 5 (14.3) | 2.66, 0.95–7.45 | 19 (54.3) | **2.19, 1.09–4.39** |
| No, 424 (92.4) | 13 (3.1) | Reference | 25 (5.9) | Reference | 149 (35.1) | Reference |
| Patronized female sex worker | | | | | | |
| Yes, 327 (76.2 | 12 (3.7) | 1.90, 0.42–8.65 | 23 (7.0) | 1.47, 0.54–3.96 | 114 (34.9) | 0.90, 0.56–1.47 |
| No, 102 (23.8) | 2 (2.0) | Reference | 5 (4.9) | Reference | 38 (37.3) | Reference |
| Had sex with men or boys | | | | | | |
| Yes, 125 (28.4) | 4 (3.2) | 1.01, 0.31–3.28 | 10 (8.0) | 1.35, 0.61–3.00 | 55 (44.0) | **1.59, 1.04–2.44** |
| No, 315 (71.6) | 10 (3.2) | Reference | 19 (6.0) | Reference | 104 (33.0) | Reference |
| Incarcerated | | | | | | |
| Yes, 264 (57.1) | 7 (2.7) | 0.74, 0.26–2.15 | 17 (6.4) | 0.98, 0.46–2.07 | 94 (35.6) | 0.89, 0.61–1.30 |
| No, 198 (42.9) | 7 (3.5) | Reference | 13 (6.6) | Reference | 76 (38.4) | Reference |
| Injected in prison | | | | | | |
| Yes, 80 (17.4) | 5 (6.3) | 2.75, 0.90–8.43 | 10 (12.5) | **2.57, 1.15–5.73** | 33 (41.3) | 1.27, 0.78–2.08 |
| No, 183 (82.6) | 9 (2.4) | Reference | 20 (5.3) | Reference | 135 (35.5) | Reference |
| Had multiple incarcerations | | | | | | |
| Yes, 54 (11.8) | 3 (5.6) | 2.11, 0.57–7.80 | 6 (11.1) | 1.98, 0.77–5.10 | 23 (42.6) | 1.33, 0.75–2.37 |
| No, 405 (88.2) | 11 (2.7) | Reference | 24 (5.9) | Reference | 145 (35.8) | Reference |

*HBsAg, hepatitis B surface antigen; HCV, hepatitis C virus; OR, odds ration; CI, confidence interval; IQR, interquartile range (25%–75%). **Boldface** values are statistically significant.

†Iranian, Pakistani, and Tajikistanian.

‡Unmarried, divorced, or widowed.

§1 US dollar = 49 Afghanis.
